# Evolving therapeutic strategies in IgA nephropathy: from conservative management to targeted interventions

**DOI:** 10.5339/qmj.2024.22

**Published:** 2024-09-23

**Authors:** Essa Abuhelaiqa, Mohamad M. Alkadi, Omar Fituri

**Affiliations:** 1 Division of Nephrology, Department of Medicine, Hamad Medical Corporation, Doha, Qatar *Email: malkadi@hamad.qa

Immunoglobin A nephropathy (IgAN) is the most common glomerulonephritis in adults worldwide.^1^ This immune-mediated glomerular disease progresses insidiously to end-stage kidney disease (ESKD) in 50%–80% of patients over 10–20 years and is often diagnosed at late stages.^2^ The pathogenesis of IgAN has been theorized by the “four-hit” model centered on the increased circulating levels of galactose-deficient IgA1 antibodies. Despite advancements, no commercially available antibody testing is sufficiently sensitive or specific to diagnose IgAN, making kidney biopsy the gold standard for diagnosis.^3^

Patients with IgAN and proteinuria >0.5 g/day have a 9.1-fold increase in the progression of kidney disease compared to those with <0.5 g/day proteinuria.^4,5^ Thus, proteinuria serves as a critical surrogate marker for IgAN progression. Up until recently, the management of IgA nephropathy has been primarily conservative. The 2021 Kidney Disease: Improving Global Outcomes (KDIGO) clinical practice guidelines recommend lifestyle modifications, including physical exercise, weight control, sodium restriction, and blood pressure control. Additionally, the guidelines recommend using a renin-angiotensin-aldosterone system inhibitor (RAASi), such as angiotensin II receptor blockers (ARB) or angiotensin-converting enzyme inhibitors (ACEI) in patients with proteinuria.^6^

Sodium-glucose cotransporter-2 inhibitors (SGLT2i) have revolutionized the management of chronic kidney disease (CKD) patients with type 2 diabetes mellitus. The nephroprotective benefits were beyond glycemic reduction, as dapagliflozin in patients with chronic kidney disease (DAPA-CKD) and empagliflozin in patients with chronic kidney disease (EMPA-Kidney) trials demonstrated significant reductions of proteinuria and kidney disease progression in non-diabetic CKD patients with albuminuria.^7,8^ Interestingly, a pre-specified subgroup analysis of 270 IgAN patients in the DAPA-CKD trial showed a significant reduction of a primary composite endpoint of sustained decline in estimated glomerular filtration rate (eGFR) ≥ 50%, ESKD or death by 71% in the SGLT2i group compared to the placebo group. However, there was no run-in period in the trial to optimize conservative care of IgAN, most patients had baseline chronic kidney disease (mean eGFR of 43.8 mL/min/1.73 m^2^) and were relatively old (mean age of 51.2 years).^9^ Therefore, further studies are still required to evaluate the benefits in patients with IgAN, especially those with eGFR ≥ 90 mL/min/1.73 m^2^.

Sparsentan, a dual endothelin A receptor antagonist, and ARB with both hemodynamic and anti-inflammatory properties showed a 40% reduction in proteinuria compared to irbesartan in the efficacy and safety of sparsentan versus irbesartan in patients with IgA nephropathy (PROTECT) trial. Furthermore, patients in the sparsentan group had a slower rate of eGFR decline than those in the irbesartan group.^10^ This led to an accelerated United States Food and Drug Administration (FDA) approval of sparsentan in IgAN in 2023. While the PROTECT trial did not address the effects of adding SGLT2i, the authors have initiated an extension study to add SGLT2i.

The use of immunosuppressive medications in IgAN has been controversial. The Intensive Supportive Care plus Immunosuppression in IgAN (STOP-IgAN) trial showed that the addition of immunosuppression (corticosteroids, cyclophosphamide, and azathioprine) to intensive supportive care in patients with IgAN and proteinuria > 0.75g/d was associated with more adverse effects with no significant short-term or long-term renal benefits.^11,12^ In 2022, “The Effect of Oral Methylprednisolone on Decline in Kidney Function or Kidney Failure in Patients with IgA Nephropathy: The TESTING Randomized Clinical Trial” showed that the use of 0.6–0.8 mg/kg/day of methylprednisolone in IgAN patients with > 1 g/day proteinuria significantly reduced the composite outcome of kidney function decline, kidney failure, or death due to kidney disease by 40% compared to placebo. However, due to the excess of serious infections in the steroids group (9% vs. 0.8%; *p* < 0.003), the trial had to be modified to use a lower dose of methylprednisolone (0.4 mg/kg/day) and antibiotic prophylaxis and continued to show similar benefits in kidney outcomes (hazard ratio (HR) 0.27, 95% CI 0.11–0.65; *p* < 0.004) with no increased risk of infection (4% vs. 2%; *p* = 0.45).^13^ This led to the introduction of Nefecon, an enteric-coated budesonide that delivers corticosteroids directly to the terminal ilium and targets gut-associated lymphoid tissue. “The Effect of Nefecon in Patients with Primary IgA Nephropathy at Risk of Developing End-Stage Renal Disease (NefIgArd)” trial showed significant proteinuria reduction in the Nefecon group compared to the placebo (31% vs. 5%, *p* < 0.0001) and eGFR decline (0.17 mL/min vs. 4.04 mL/min, *p* = 0.001) within 9 months of therapy with no significant increased risk of infection.^14^ The galactose-deficient IgA1 antibody levels were also significantly reduced in the nefecon group (*p* = 0.015). As a result, the FDA has issued an accelerated approval for Nefecon to treat IgAN in 2021 and full approval in 2023 after the extension trial showed sustained benefits at 24 months.^15^ However, proteinuria increased again at month 12, and eGFR decline started to run parallel in the two groups after stopping Nefecon at month 9. This might indicate the waning of its effect and the possible need for repeated courses. In addition, the cost of Nefecon is undeniably high, and it is unclear whether Nefecon is superior to the older and cheaper enteric-coated budesonide preparations developed for inflammatory bowel disease or low-dose steroids. Lastly, the impact of SGLT2i on kidney outcomes, both with and without Nefecon, has yet to be evaluated.

The role of mycophenolate mofetil (MMF) in treating IgAN has yet to be established due to conflicting results. Older studies showed no benefit,^16^ however, a more recent randomized study in the Chinese population showed that 18-month treatment with MMF reduced the risk of a composite outcome of doubling serum creatinine, ESKD, or death due to kidney or cardiovascular disease by 77% after 3 years of follow-up.^17^ Thus, the 2021 KDIGO guidelines recommend using MMF as a steroid-sparing agent in Chinese patients only.^4^

Complement inhibition offers a promising therapeutic approach by targeting C3, C5, C5a, and factor B to prevent glomerular injury during the fourth “hit,” with encouraging results in reducing proteinuria. Iptacopan, a monoclonal factor B inhibitor that blocks the complement alternative pathway, recently received FDA accelerated approval in August 2024. Iptacopan reduced proteinuria by 38% at 9 months of therapy compared to placebo (*p* < 0.0001).^18,19^ Remarkably, authors have adjusted for ethnicity, age, chronicity of disease and SGLT2i. While the finalized results of the trial are yet to be published, the interim results are promising and emphasize the importance of targeting the complement pathway in IgAN.

Other emerging IgAN therapies are focused on mechanistic targeting of the disease origin, such as B-cell-activating factor (BAFF) and A proliferation-inducing ligand (APRIL) signal inhibition, which has been promising as they target the first “hit."^20^ Medications such as belimumab, telitacicept, and atacicept are entering phase II trials. Sibeprenlimab, an APRIL-specific monoclonal antibody, received breakthrough FDA approval in February 2024 and is currently in a phase III trial after passing its safety profile. Sibeprenlimab showed a dose-dependent significant reduction in proteinuria and eGFR decline compared to placebo, likely related to a decreased level of galactose-deficient IgA1 antibodies and APRIL emphasizing the mechanistic benefit of the drug.^21^ The decreased APRIL levels were not sustained after sibeprenlimab discontinuation, likely indicating that long-term treatment might be required, increasing the risk of antibody development against sibeprenlimab and therapy resistance. Furthermore, the effect of SGLT2i is not addressed in the current study.

The future of IgAN therapy is exciting, as disease management is departing beyond conservative management to the realm of early disease intervention with targeted immunosuppression and protection of further injury with hemodynamic, anti-fibrotic, and anti-inflammatory drugs. A multitargeted drug approach is the likely path to treating IgAN; therefore, gaps in the combination therapy approach need to be addressed in future studies. Diagnostic and therapeutic biomarkers are also required for early diagnosis and personalized disease management.

## References

[1] McGrogan A, Franssen CF, de Vries CS (2011;). The incidence of primary glomerulonephritis worldwide: a systematic review of the literature. Nephrol Dial Transplant.

[2] Alkadi MM, Abuhelaiqa EA, Thappy SB (2022). Kidney biopsy in patients with markedly reduced kidney function. Kidney Int Rep.

[3] Ohyama Y, Renfrow MB, Novak J (2021). Aberrantly glycosylated IgA1 in IgA nephropathy: what we know and what we don’t know. J Clin Med.

[4] Le W, Liang S, Hu Y (2012;). Long-term renal survival and related risk factors in patients with IgA nephropathy: results from a cohort of 1155 cases in a Chinese adult population. Nephrol Dial Transplant.

[5] Thompson A, Carroll K, A Inker L (2019). Proteinuria reduction as a surrogate end point in trials of IgA nephropathy. Clin J Am Soc Nephrol.

[6] Rovin BH, Adler SG, Barratt J (2021;). Executive summary of the KDIGO 2021 guideline for the management of glomerular diseases. Kidney Int.

[7] Heerspink HJL, Stefansson BV, Correa-Rotter R (2020;). Dapagliflozin in patients with chronic kidney disease. N Engl J Med.

[8] Herrington WG, Staplin N, The E-KCG (2023;). Empagliflozin in patients with chronic kidney disease. N Engl J Med.

[9] Wheeler DC, Toto RD, Stefánsson BV (2021). A pre-specified analysis of the DAPA-CKD trial demonstrates the effects of dapagliflozin on major adverse kidney events in patients with IgA nephropathy. Kidney Int.

[10] Rovin BH, Barratt J, Heerspink HJL (2023). Efficacy and safety of sparsentan versus irbesartan in patients with IgA nephropathy (PROTECT): 2-year results from a randomised, active-controlled, phase 3 trial. Lancet.

[11] Rauen T, Eitner F, Fitzner C, Sommerer C, Zeier M, Otte B, Panzer U, Peters H, Benck U, Mertens PR, Kuhlmann U, Witzke O, Gross O, Vielhauer V, Mann JF, Hilgers RD, Floege J, STOP-IgAN Investigators (2015). Intensive supportive care plus Immunosuppression in IgA nephropathy. N Engl J Med.

[12] Rauen T, Wied S, Fitzner C, Eitner F, Sommerer C, Zeier M, Otte B, Panzer U, Budde K, Benck U, Mertens PR, Kuhlmann U, Witzke O, Gross O, Vielhauer V, Mann JFE, Hilgers RD, Floege J, STOP-IgAN Investigators (2020). After ten years of follow-up, no difference between supportive care plus immunosuppression and supportive care alone in IgA nephropathy. Kidney Int.

[13] Lv J, Wong MG, Hladunewich MA, Jha V, Hooi LS, Monaghan H, Zhao M, Barbour S, Jardine MJ, Reich HN, Cattran D, Glassock R, Levin A, Wheeler DC, Woodward M, Billot L, Stepien S, Rogers K, Chan TM, Liu ZH, Johnson DW, Cass A, Feehally J, Floege J, Remuzzi G, Wu Y, Agarwal R, Zhang H, Perkovic V, TESTING Study Group (2022). Effect of oral methylprednisolone on decline in kidney function or kidney failure in patients with IgA nephropathy: the TESTING randomized clinical trial. JAMA.

[14] Barratt J, Lafayette R, Kristensen J (2023). Results from part A of the multi-center, double-blind, randomized, placebo-controlled NefIgArd trial, which evaluated targeted-release formulation of budesonide for the treatment of primary immunoglobulin A nephropathy. Kidney Int.

[15] Lafayette R, Kristensen J, Stone A (2023). Efficacy and safety of a targeted-release formulation of budesonide in patients with primary IgA nephropathy (NefIgArd): 2-year results from a randomised phase 3 trial. Lancet.

[16] Peng XJ, Zheng WM, Fu R, Huang YH, Deng MH, Tao SS, Wang TJ, Zhu C (2021). Efficacy and safety of mycophenolate mofetil in the treatment for IgA nephropathy: a meta-analysis of randomized controlled trials. Clin Exp Nephrol.

[17] Hou FF, Xie D, Wang J, Xu X, Yang X, Ai J, Nie S, Liang M, Wang G, Jia N, MAIN Trial Investigators (2023). Effectiveness of mycophenolate mofetil among patients with progressive IgA nephropathy: a randomized clinical trial. JAMA Netw Open.

[18] Zhang H, Rizk DV, Perkovic V, Maes B, Kashihara N, Rovin B, Trimarchi H, Sprangers B, Meier M, Kollins D, Papachristofi O, Milojevic J, Junge G, Nidamarthy PK, Charney A, Barratt J (2024). Results of a randomized double-blind placebo-controlled Phase 2 study propose iptacopan as an alternative complement pathway inhibitor for IgA nephropathy. Kidney Int.

[19] Perkovic V, Kollins D, Papachristofi O (2024). Efficacy and safety of iptacopan in patients with primary IgA nephropathy: interim analysis results of the Phase 3 APPLAUSE-IgAN study. Nephrology Dialysis Transplantation.

[20] El Karoui K, Fervenza FC, De Vriese AS (2024). Treatment of IgA nephropathy: a rapidly evolving field. J Am Soc Nephrol.

[21] Mathur M, Barratt J, Chacko B (2024). A phase 2 trial of sibeprenlimab in patients with IgA nephropathy. N Engl J Med.

